# EBV persistence without its EBNA3A and 3C oncogenes in vivo

**DOI:** 10.1371/journal.ppat.1007039

**Published:** 2018-04-30

**Authors:** Anita Murer, Donal McHugh, Nicole Caduff, Jens Kalchschmidt, Mario Barros, Andrea Zbinden, Riccarda Capaul, Gerald Niedobitek, Martin Allday, Obinna Chijioke, Christian Münz

**Affiliations:** 1 Viral Immunobiology, Institute of Experimental Immunology, University of Zürich, Zürich, Switzerland; 2 Genomics and Immunity, NIAMS, National Institutes of Health, Bethesda, MD, United States of America; 3 Institute of Pathology, Unfallkrankenhaus Berlin, Berlin, Germany; 4 Institute of Medical Virology, University of Zürich, Zürich, Switzerland; 5 Molecular Virology, Department of Medicine, Imperial College London, London, United Kingdom; 6 Institute of Pathology and Molecular Pathology, University Hospital Zürich, Zürich, Switzerland; University of Pennsylvania Medical School, UNITED STATES

## Abstract

The oncogenic Epstein Barr virus (EBV) infects the majority of the human population and usually persists within its host for life without symptoms. The EBV oncoproteins nuclear antigen 3A (EBNA3A) and 3C (EBNA3C) are required for B cell transformation in vitro and are expressed in EBV associated immunoblastic lymphomas in vivo. In order to address the necessity of EBNA3A and EBNA3C for persistent EBV infection in vivo, we infected NOD-*scid* γ_c_^null^ mice with reconstituted human immune system components (huNSG mice) with recombinant EBV mutants devoid of EBNA3A or EBNA3C expression. These EBV mutants established latent infection in secondary lymphoid organs of infected huNSG mice for at least 3 months, but did not cause tumor formation. Low level viral persistence in the absence of EBNA3A or EBNA3C seemed to be supported primarily by proliferation with the expression of early latent EBV gene products transitioning into absent viral protein expression without elevated lytic replication. In vitro, EBNA3A and EBNA3C deficient EBV infected B cells could be rescued from apoptosis through CD40 stimulation, mimicking T cell help in secondary lymphoid tissues. Thus, even in the absence of the oncogenes EBNA3A and 3C, EBV can access a latent gene expression pattern that is reminiscent of EBV persistence in healthy virus carriers without prior expression of its whole growth transforming program.

## Author summary

Epstein Barr virus (EBV) was discovered 54 years ago as the first human candidate tumor virus. In vitro, EBV can readily transform human B cells into indefinitely growing cell lines. In this transformation process, multiple EBV proteins play an important role, such as the two oncogenes EBNA3A and especially EBNA3C. To address the necessity of these oncogenes in vivo, we investigated EBNA3A and 3C deficient EBV for their persistence in mice with reconstituted human immune system components (huNSG mice), an in vivo model for EBV infection, associated tumorigenesis and immune control. Here, we show that EBV devoid of EBNA3A or EBNA3C was able to establish latent infection in vivo. The persistence was characterized through proliferation with the expression of early latent EBV gene products transitioning into absent viral protein expression, but without tumor formation. Furthermore, we were able to rescue in vitro infected B cells with EBNA3A or EBNA3C deficient EBV from apoptosis by mimicking T cell help. This might allow the virus to access the gene expression pattern that is thought to also mediate persistence in healthy EBV carriers and does not seem to require prior expression of all growth transforming viral proteins.

## Introduction

Epstein Barr virus (EBV) was discovered as the first human candidate tumor virus in Burkitt lymphoma 54 years ago [1, 2]. EBV is probably the most potent of all the human tumor viruses, because it can readily transform human B cells in vitro [3]. In the resulting lymphoblastoid cell lines (LCLs) all nine latent EBV proteins and non-translated RNAs are expressed [4]. These include the six nuclear antigens (EBNA1, 2, LP, 3A-C) and the three latent membrane proteins (LMP1, 2A and 2B). This so-called latency III program can also be found in naïve B cells of healthy virus carriers and lymphoproliferations that emerge in immune compromised individuals, like post-transplant lymphoproliferative disorder (PTLD) and immunoblastic lymphoma [5]. In germinal center B cells and latency II associated malignancies, like classical Hodgkin lymphoma, only the EBNA1, LMP1 and LMP2 proteins are present [4, 5]. Latency I is characterized by the sole expression of EBNA1 and this viral expression pattern can be found in Burkitt lymphomas and EBV infected homeostatically proliferating memory B cells [6]. Finally, EBV persists in non-replicating memory B cells without viral protein expression and reactivates upon plasma cell differentiation to produce infectious viral particles at mucosal surfaces for transmission [7, 8]. These previously published studies suggested that EBV persistence can only be achieved upon further differentiation of latency III infected naïve B cells through latency II and I towards memory B cells in which the virus can reside undetectable to the immune system.

Accordingly, EBV latency III establishment during primary B cell infection in vitro and the resulting outgrowth of LCLs were taken as surrogates for EBV persistence. Upon B cell infection by EBV, the transient expression of the two viral Bcl-2 homologues BHRF1 and BALF1 initially ensures the survival of EBV harboring cells [9]. During the first two days, proliferation of the infected cells is initiated by EBNA2, probably with the help of EBNA-LP, via expression of cell cycle genes including c-myc, cyclin D2 and E [10]. The resulting DNA damage response and BIM as well as p16^INK4a^ up-regulation is blocked primarily by EBNA3C with the help of EBNA3A [10–13]. Therefore, latency III expressing lymphoblastoid cell lines cannot be established with EBV deficient in EBNA3C and only with great difficulties from EBNA3A knock-out viruses [14, 15]. Two weeks after initial infection LMP1, 2A and 2B expression will start to contribute to the proliferation of EBV infected B cells via LMP1-dependent NF-κB activation, mimicking CD40 signaling, and LMP2A and 2B’s survival-inducing B cell receptor-like signaling [16, 17]. In order for the latency III transcription program not to kill the host due to uncontrolled B cell proliferation, EBNA3B allows for the increased expression of chemokines that attract T cells, which then in turn restrict latency III transformed B cells by their cytotoxic function and promote the switch from latency III to II by repressing EBNA2 via germinal center associated cytokines [18–21]. These studies postulated that EBNA3C is essential for latency III establishment and that B cell transformation is essential for EBV persistence in vivo.

Therefore, we investigated EBNA3A and EBNA3C deficient EBV viruses for their persistence in mice with reconstituted human immune system components (huNSG mice), an in vivo model for persistent EBV infection, EBV associated lymphomagenesis and cell-mediated immune control of EBV [22]. Surprisingly, we found that both EBNA3A and EBNA3C deficient EBVs were able to persist in huNSG mice, albeit at a lower level compared to wildtype infection. The presence of the mutant viruses could mainly be detected in secondary lymphoid tissues without lymphomagenesis after four to twelve weeks of infection. The persistence was characterized by EBER and EBNA2 expression accompanied by very low LMP1 expression, which transited into EBER only expression after three months. Especially EBNA3C deficient EBV showed markedly reduced or even absent LMP1 expression. Supplementation of CD40 signaling rescued EBNA3C deficient EBV infected B cells in vitro. Thus, we suggest that EBV can persist without EBNA3A or EBNA3C expression in vivo, utilizing signals from the microenvironment of secondary lymphoid tissues, including CD40 stimulation by CD40L. With this microenvironmental help EBV seems to be able to access latency 0 after EBNA2 driven proliferation without ever expressing all proteins associated with the latency III program. This suggests that latency III and 0 are not consecutive EBV gene expression programs, but alternative pathways after EBNA2 driven proliferation.

## Results

### EBV persists without EBNA3A or EBNA3C in vivo

**Fig 1:**
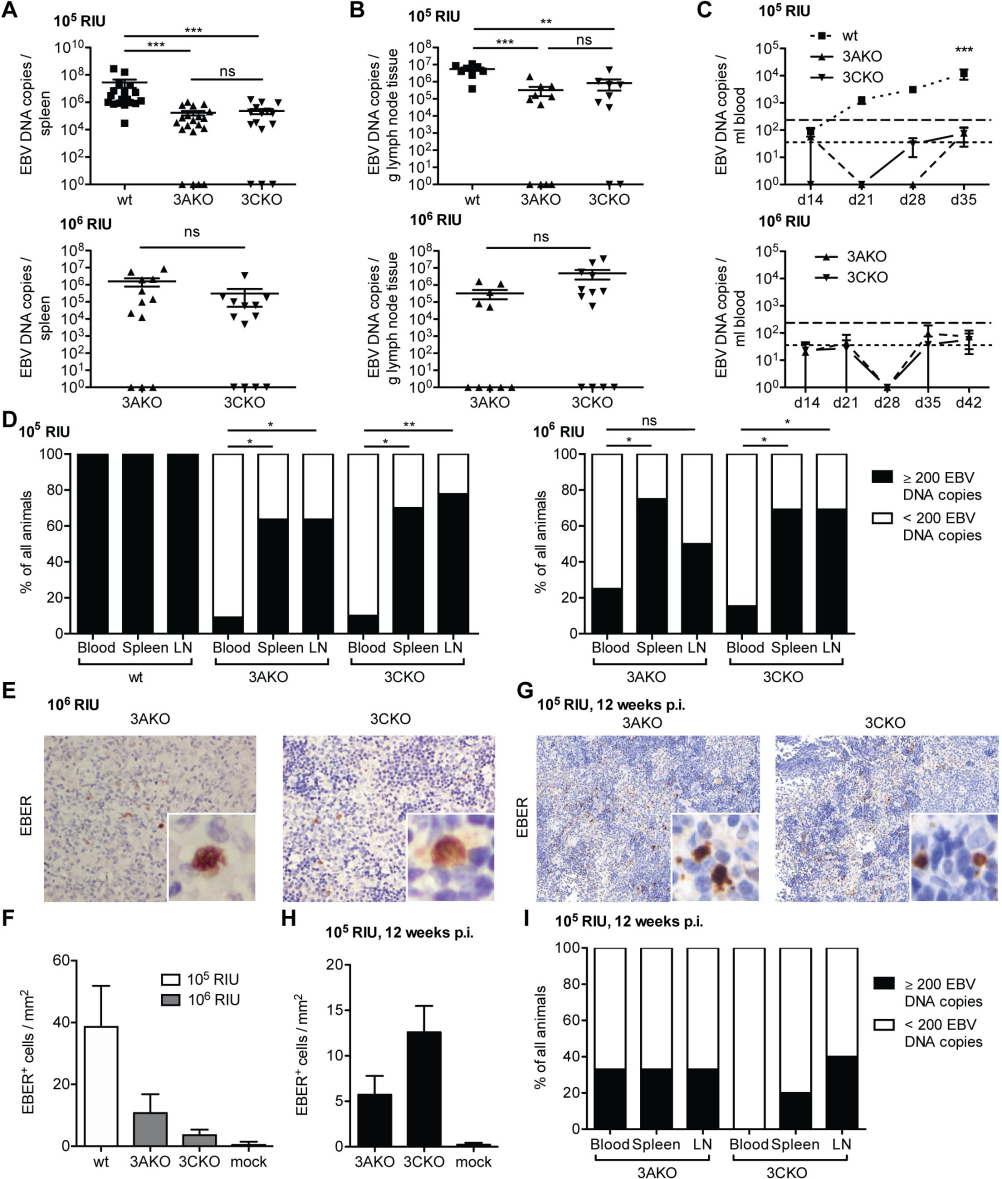
EBV persists without EBNA3A or EBNA3C in vivo. **(A)** Splenic endpoint viral DNA load and **(B)** viral DNA load per gram lymph node tissue determined by qPCR of huNSG mice infected with either 10^5^ RIU of wt, 3AKO or 3CKO for 5 weeks (spleen: n = 18-21/group, lymph node: n = 8-11/group) or 10^6^ RIU of 3AKO or 3CKO for 6 weeks (spleen: n = 13-14/group, lymph node: n = 12-13/group). Values for mice in which no viral DNA was detected are plotted on the X-axis. **(C)** Blood DNA viral load over time determined by qPCR of huNSG mice infected with either 10^5^ RIU of wt, 3AKO or 3CKO 5 weeks p.i. (n = 18-21/group) or 10^6^ RIU of 3AKO or 3CKO 6 weeks p.i. (n = 13-14/group). Horizontal dashed line indicates the viral load of 3 times the lower limit of quantification (LLOQ). Horizontal dotted line indicates the LLOQ. **(D)** Frequency of huNSG mice infected with 10^5^ RIU of 3AKO, 3CKO or wt 5 weeks p.i. or 10^6^ RIU of 3AKO or 3CKO 6 weeks p.i. with EBV DNA copies above (≥200) or below (<200) 3 times the LLOQ in either the blood, the spleen or lymph nodes (n = 10-13/group) as determined by qPCR. Pooled data from 4 low and 2 high infectious dose experiments. *P < 0.05, **P < 0.01, Fisher’s exact test. **(E)** EBER-ISH (original magnification, 400x) of splenic sections from huNSG mice infected with 10^6^ RIU of 3AKO or 3CKO 6 weeks p.i.. **(G)** EBER-ISH (original magnification, 200x) of splenic sections from huNSG mice infected with 10^5^ RIU of 3AKO or 3CKO 12 weeks p.i.. **(F, H)** Quantification of EBER^+^ cells/mm^2^ of **E** (n = 10-13/group) and **G** (n = 3-5/group) respectively and of huNSG mice infected with 10^5^ RIU of wt 5 weeks p.i. (n = 4/group) or mock (n = 3-8/group). **(I)** Frequency of huNSG mice infected with 10^5^ RIU of 3AKO or 3CKO 12 weeks p.i., with EBV DNA copies above (≥200) or below (<200) 3 times the LLOQ in either the blood, the spleen or lymph nodes (n = 3-5/group) as determined by qPCR. Pooled data from 2 experiments. **(A-C)** Pooled data from 4 low and 2 high infectious dose experiments are displayed with mean ± SEM. **P < 0.01, ***P < 0.001, two-way ANOVA with Bonferroni correction for blood viral load and Mann-Whitney U test for splenic viral load, lymph node viral load and EBER-ISH.

EBNA3A knock-out EBV (3AKO) and EBNA3C knock-out EBV (3CKO) were previously shown to be unable to establish indefinitely growing LCLs in vitro [14], and only few EBNA3A deficient LCLs were derived with significant difficulties [15]. Until now, the importance of EBNA3A and EBNA3C for EBV persistence in vivo has not been investigated. Our group among others could previously demonstrate that NOD-*scid* γ_c_^null^ mice with reconstituted human immune system compartments (huNSG mice) are suitable models to study persistent EBV infection and immune control in vivo [23, 24]. In order to identify the role of EBNA3A or EBNA3C during EBV infection in vivo we inoculated huNSG mice with 10^5^ Raji-infectious units (RIU) of 3AKO, 3CKO or wildtype EBV (wt). EBV DNA was readily detected in the spleen and lymph nodes of the majority of both 3AKO and 3CKO infected mice 5 weeks p.i. (Fig 1A and 1B). The viral DNA burden, however, was significantly lower compared to that of wt infected animals (Fig 1A and 1B). In order to detect persistent EBV infection in the absence of EBNA3A and EBNA3C more clearly, huNSG mice were infected with a higher inoculation dose of 10^6^ RIU in subsequent experiments. Wt infection with 10^6^ RIU results in rapid weight loss and death of the infected animals and was thus not performed. The resulting viral DNA loads in the spleen and lymph nodes were slightly higher compared to the 10^5^ RIU infections and remained similar between the two KO virus infected groups (Fig 1A and 1B). However, despite infection with the higher infectious dose, viral DNA in the blood of 3AKO or 3CKO infected animals could still only rarely be detected at very low levels (Fig 1C). Hence, persistence of 3AKO or 3CKO viruses was mainly found in secondary lymphoid organs of infected animals (Fig 1D). Active EBV transcription demonstrated by EBER in situ hybridization in splenic sections from 3AKO and 3CKO infected animals confirmed viral persistence (Fig 1E). The number of EBER^+^ cells/mm^2^ in the spleen was similar in 3AKO and 3CKO, but reduced compared to wt infected mice (Fig 1F). EBER^+^ cells could be found up to 3 months of infection (Fig 1G and 1H) and likewise, EBV DNA was still detected in spleen and lymph nodes of 3AKO or 3CKO infected mice 3 months p.i. (Fig 1I). EBV infected cells, illustrated by EBNA2 staining or EBER in situ hybridization, were mostly CD20^+^ and CD3^-^ indicating viral presence in B cells in all experimental groups (S1 Fig). Despite persistent low-level infection, neither 3AKO nor 3CKO inoculation resulted in tumor formation in huNSG mice. Hence, in contrast to previously published in vitro data [14], EBNA3A and EBNA3C seem to be dispensable for persistent EBV infection in vivo.

### EBNA3A and EBNA3C deficient EBV infection causes CD8^+^ T cell expansion

**Fig 2:**
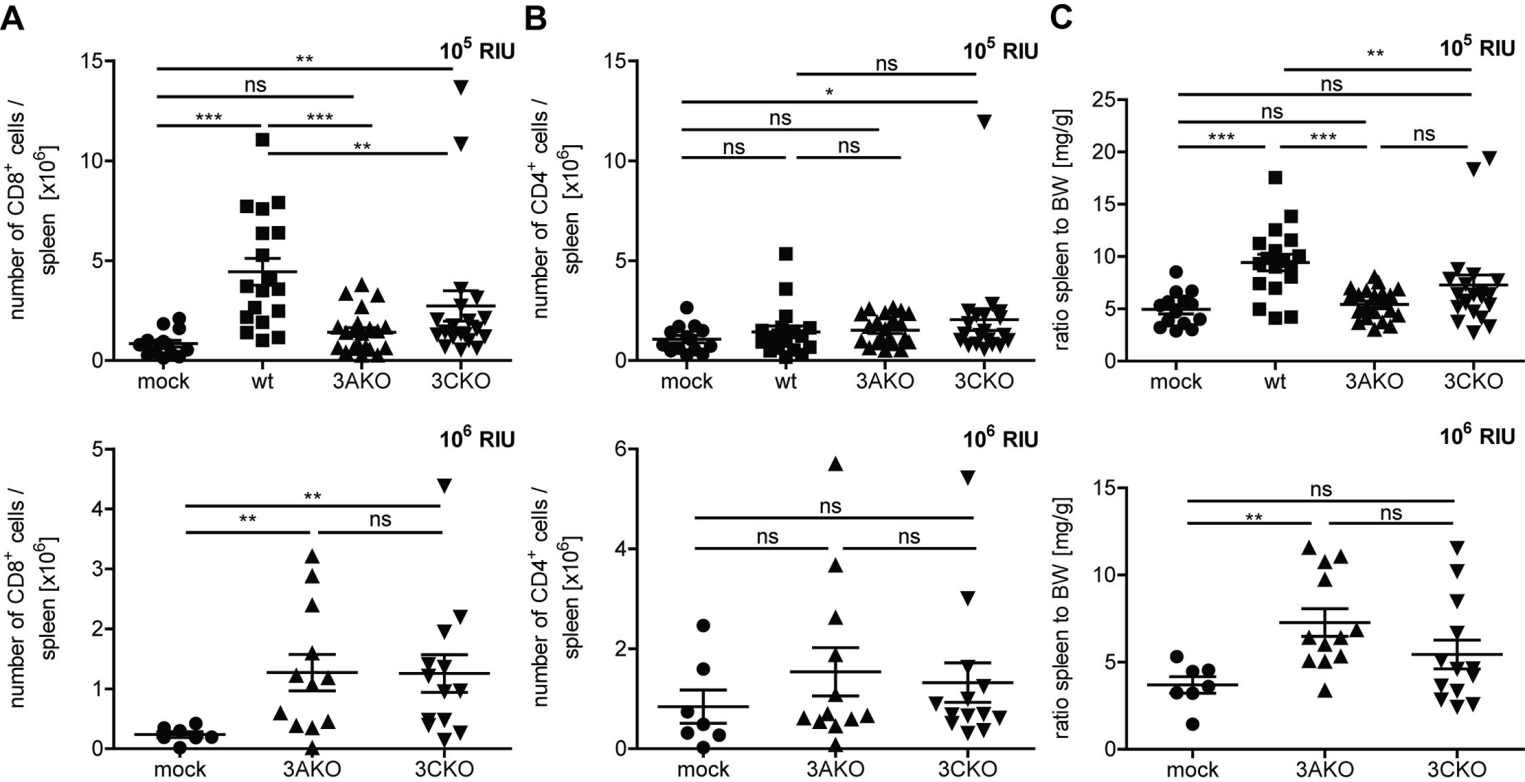
EBNA3A or EBNA3C deficient EBV infection causes CD8^+^ T cell expansion. **(A)** The number of splenic CD8^+^ T cells and **(B)** splenic CD4^+^ T cells of huNSG mice infected with either 10^5^ RIU of wt, 3AKO or 3CKO 5 weeks p.i. (n = 14-21/group) or 10^6^ RIU of 3AKO or 3CKO 6 weeks p.i. (n = 7-13/group) or non-infected control (mock) huNSG mice was determined by flow cytometry, applying the determined frequency to the spleen cell count. **(C)** The ratio of spleen to body weight (BW) of individual huNSG mice infected with either 10^5^ RIU of wt, 3AKO or 3CKO 5 weeks p.i. (n = 14-21/group) or 10^6^ RIU of 3AKO or 3CKO 6 weeks p.i. (n = 7-13/group) or non-infected control (mock) huNSG mice is depicted. **(A-C)** Pooled data from 4 low and 2 high infectious dose experiments with mean ± SEM. *P < 0.05, **P < 0.01, ***P < 0.001, Mann-Whitney U test.

Wildtype infection in huNSG mice has been shown to induce the expansion of human CD8^+^ T cells and result in splenomegaly [24]. As a second readout for EBV persistence in the absence of EBNA3A or EBNA3C, we investigated the cellular immune response in huNSG mice against 3AKO or 3CKO infection. The number of splenic (Fig 2A) and blood (S2A Fig) CD8^+^ T cells was significantly increased in 10^5^ RIU of 3CKO infected animals 5 weeks p.i. compared to uninfected animals. In low dose (10^5^ RIU) 3AKO infected mice a similar trend for an increase of splenic CD8^+^ T cells was observed (Fig 2A). However, both 3AKO and 3CKO infected mice had significantly lower numbers of splenic and blood CD8^+^ T cells compared to wt infected mice (Fig 2A and S2A Fig). High dose (10^6^ RIU) infection with 3AKO and 3CKO resulted in a significantly higher number of splenic CD8^+^ T cells in both groups compared to the uninfected group (Fig 2A) and similar trends were observed in the blood (S2A Fig). In parallel CD8^+^ T cell activation, as assessed by HLA-DR up-regulation, was observed in spleen and blood after wt and knock-out virus infections (S3A, 3B, 3C and 3D Fig). However, in contrast to CD8^+^ T cells, the number of splenic and blood CD4^+^ T cells did not differ significantly in the different experimental groups irrespective of viral dose, except for a significant splenic CD4^+^ T cell accumulation after 10^5^ RIU 3CKO infection and significant HLA-DR up-regulation on spleen and blood CD4^+^ T cells after wt infection and a trend in HLA-DR up-regulation for 3AKO and 3CKO infection (Fig 2B and S2B, S3E, S3F, S3G and S3H Figs). Furthermore, splenomegaly, characteristic for wt infection, was not observed in 3AKO or 3CKO infected mice with the lower infectious dose (Fig 2C). High dose infection with 3AKO led to significant splenomegaly compared to uninfected mice, whereas 3CKO infected mice only showed a trend towards splenomegaly (Fig 2C). Interestingly, 3AKO or 3CKO high dose infected mice had a clear increase in CD8^+^ T cells in the spleen, but to a lesser extent in the blood. These findings mirror the prevalence of 3AKO or 3CKO infected cells in secondary lymphoid tissues and indicate that cellular immune responses against 3AKO and 3CKO are mounted in huNSG mice.

### EBNA3A and EBNA3C deficient EBV persists without elevated lytic replication

**Fig 3:**
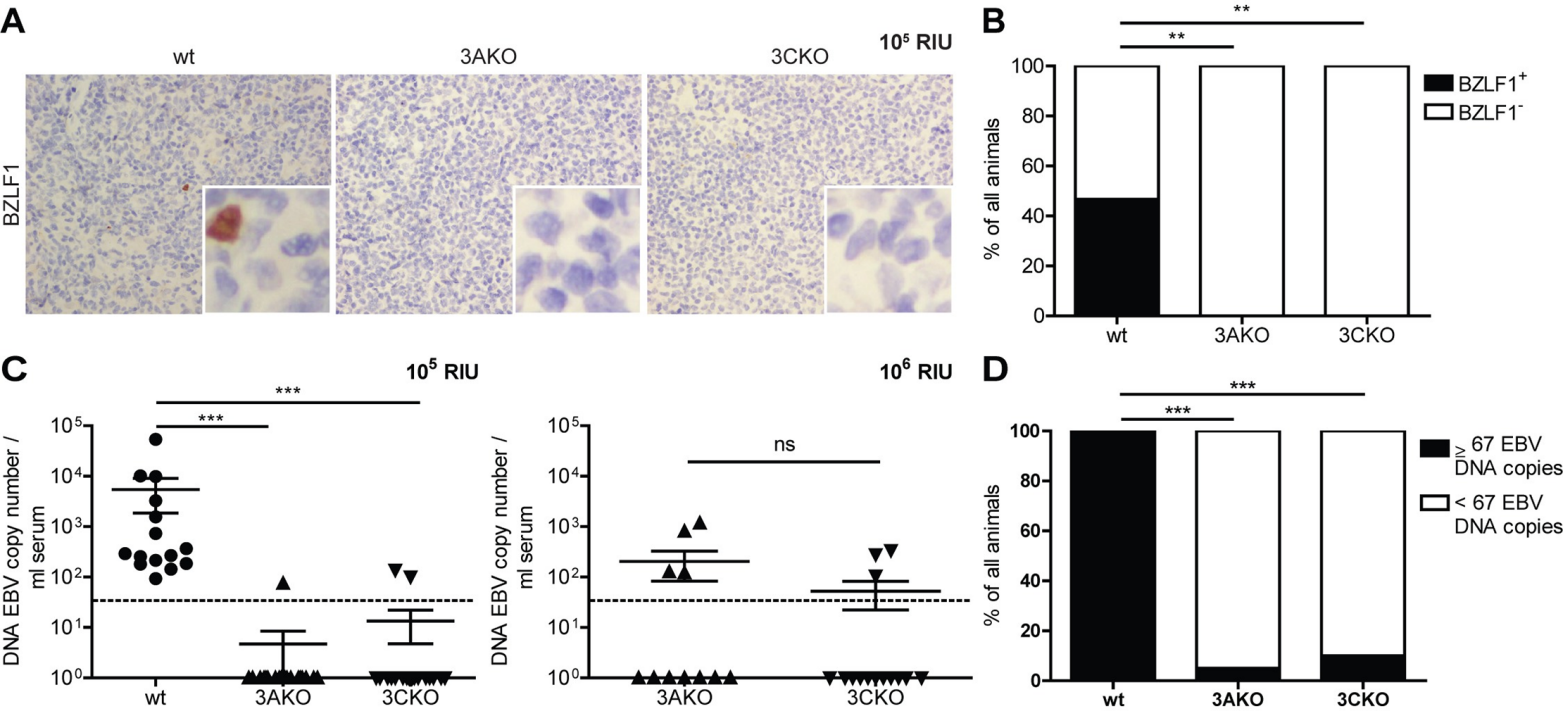
EBNA3A and EBNA3C deficient EBV persist without elevated lytic replication. **(A)** Representative immunohistochemistry staining for BZLF1 (original magnification, 400x) in splenic sections of huNSG mice infected with 10^5^ RIU of wt, 3AKO or 3CKO 5 weeks p.i.. **(B)** Frequency of huNSG mice infected with 10^5^ RIU of 3AKO, 3CKO or wt 5 weeks p.i. with BZLF1^+^ or BZLF1^-^ cells in splenic sections as determined by immunohistochemistry. **(C)** DNA EBV genome copies determined by qPCR in serum of animals infected with either 10^5^ RIU of wt, 3AKO or 3CKO 5 weeks p.i. (n = 15-20/group) or 10^6^ RIU of 3AKO or 3CKO 6 weeks p.i. (n = 11-14/group). Values for mice in which no viral DNA was detected in the serum are plotted on the X-axis. Horizontal dotted line indicates the LLOQ. Pooled data from 4 low and 2 high infectious dose experiments represented with the mean ± SEM. ***P < 0.001, Mann-Whitney U test. **(D)** Frequency of huNSG mice infected with 10^5^ RIU of 3AKO, 3CKO or wt 5 weeks p.i. with EBV DNA copies above (≥67) or below (<67) the LLOQ in the serum (n = 15-20/group) as determined by qPCR. **(B, D)** Pooled data from 4 experiments. ***P < 0.001, **P < 0.005, Fisher’s exact test.

Next, we investigated whether EBV entered into lytic replication as a means for persistence without EBNA3A or EBNA3C. BZLF1 is the first gene expressed in the lytic cycle of EBV and transactivates viral replication [25, 26], which was shown in vivo to occur in plasma cells of healthy EBV carriers [8]. Therefore, both BZLF1 expression as well as plasma cell presence was determined. The number of BZLF1^+^ cells and CD138^+^ plasma cells in splenic sections of 3AKO or 3CKO infected huNSG mice was determined by immunohistochemistry (Fig 3A and S4A and S4C Fig). BZLF1^+^ cells seemed to be absent in spleen sections of 3AKO or 3CKO infected animals, presumably due to low viral loads, since also in wt infected mice just a few BZLF1^+^ cells were present (Fig 3A and 3B, S4C Fig). Furthermore, we did not detect a significant difference in the number of plasma cells in mice infected with 3AKO or 3CKO at 10^6^ RIU compared to uninfected mice (S4A and S4B Fig). To assess lytic activity further, we measured viral DNA presence in the serum, which has previously been proposed to be correlated with the lytic phase of infection [27]. DNA viral loads seemed to be virtually absent in the serum of 3AKO and 3CKO infected animals irrespective of EBV dose, whereas in wt infected animals, DNA viral loads were observed (Fig 3C and 3D). Hence, lytic replication was mainly found in wt, but not in KO infected animals (Fig 3B and 3D). In line with these findings, we found significantly lower *BZLF1* mRNA expression in 3CKO infected B cells compared to wt infected B cells in vitro. Furthermore, there was no difference in the expression of *BZLF1* in 3AKO in comparison to wt infected B cells (S5 Fig). Together, 3AKO and 3CKO seem not to persist due to an increase in lytic replication in secondary lymphoid organs.

### EBV infected cells proliferate without EBNA3A or EBNA3C in vivo

**Fig 4:**
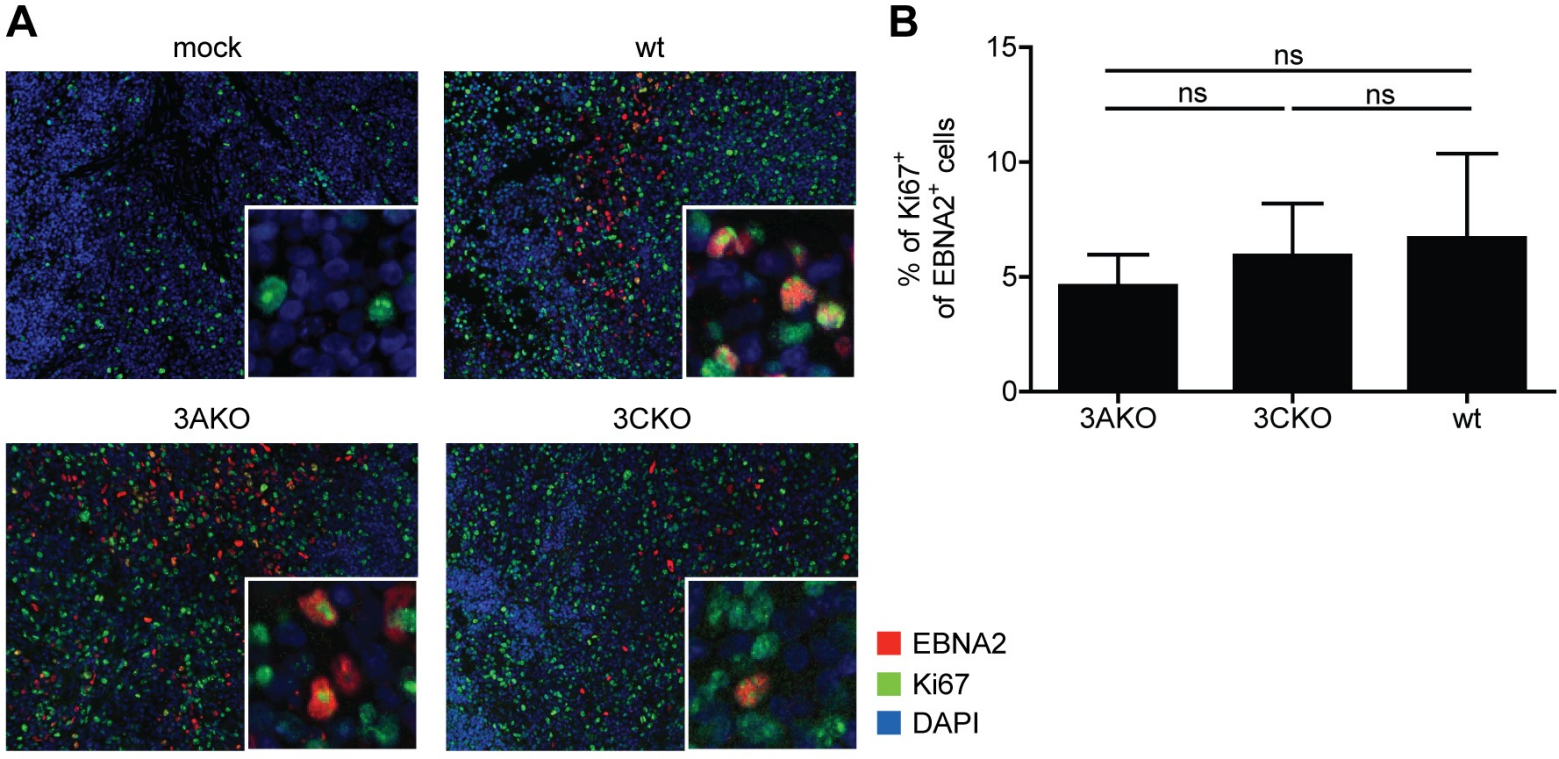
EBV infected cells proliferate without EBNA3A or EBNA3C in vivo. **(A)** Representative immunofluorescence staining for EBNA2 (red), Ki67 (green) and DAPI (blue) (original magnification, 200x) in splenic sections of huNSG mice infected with wt, 3AKO or 3CKO 5 or 6 weeks p.i.. **(B)** Quantification of **A** with the frequency of Ki67^+^ EBNA2^+^ cells of all EBNA2^+^ cells (n = 4-15/group). Pooled data from 2 experiments with mean ± SEM, Mann-Whitney U test.

In vitro 3CKO infected B cells were shown to proliferate for up to 30 days prior to eventual arrest or cell death [12, 28]. Nevertheless, the remaining living cells contain viral DNA. To investigate if the observed viral load in 3AKO or 3CKO infected mice 5 to 6 weeks p.i. was due to arrested non-proliferating B cells, we performed immunofluorescence co-stainings for EBNA2 and Ki67 of splenic sections from wt, 3AKO and 3CKO infected mice. EBNA2 positive cells were detected in most of the infected mice, but not in uninfected animals, whereas Ki67 positive cells were detected in all groups (Fig 4A). Cells double-positive for EBNA2 and Ki67 were detected in wt, as well as KO infected groups (Fig 4A). The frequency of Ki67 expressing cells of EBNA2 positive cells was comparable between wt and KO infected mice (Fig 4B). These findings reveal the proliferation of EBV infected cells in KO infected mice for up to 6 weeks.

### EBNA3A and EBNA3C deficient EBV persist without viral protein expression

**Fig 5:**
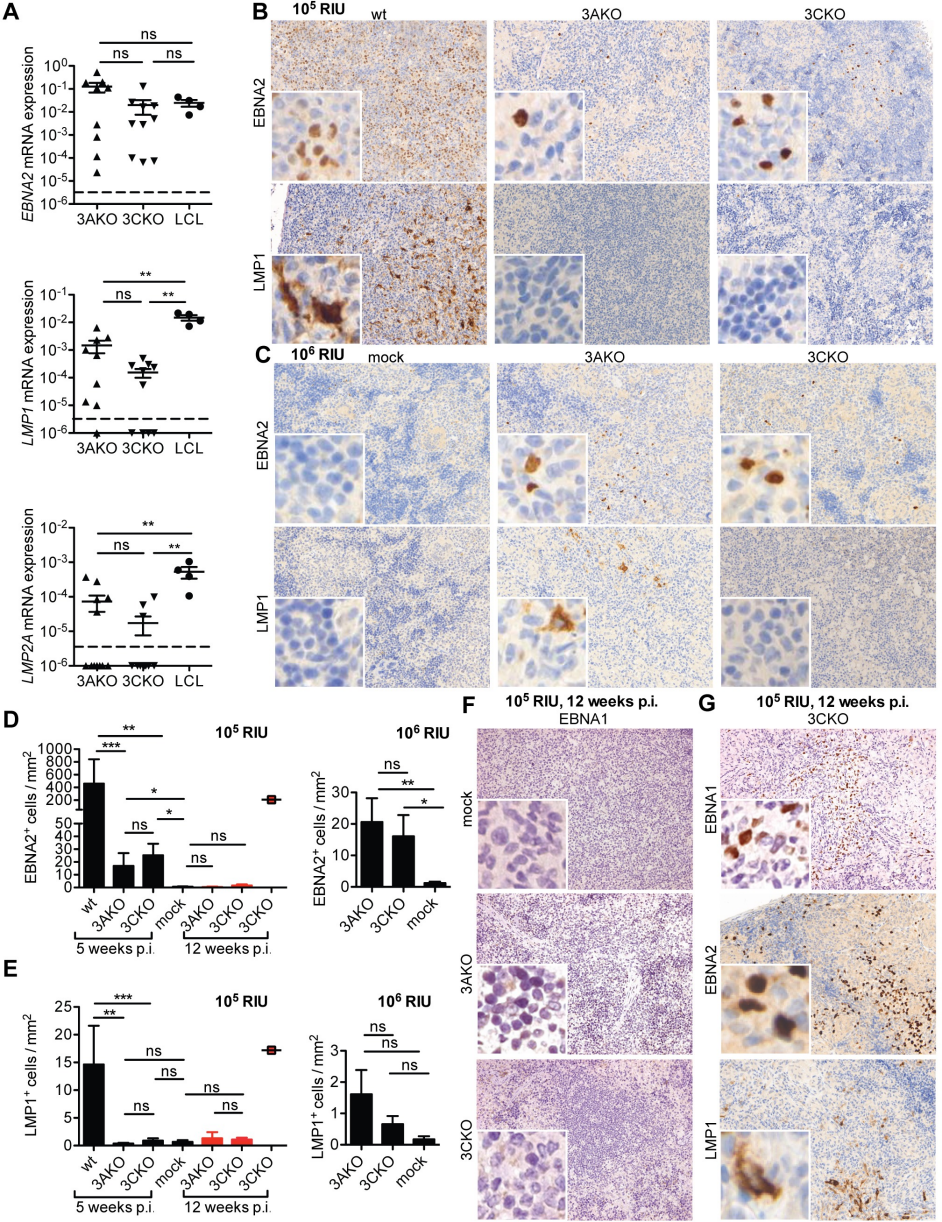
EBNA3A and EBNA3C deficient EBV persist without viral protein expression. **(A)** Relative *EBNA2*, *LMP1* and *LMP2A* mRNA expression normalized to *GAPDH* in purified splenic B cells from huNSG mice infected with 10^6^ RIU of 3AKO or 3CKO 6 weeks p.i. (n = 9-12/group) and LCL from 4 different donors. The horizontal dashed lines indicate the detection limits of the RT-qPCR assays. Values for mice in which no *LMP1* transcripts were detected are plotted on the X-axis. **(B, C, G)** Staining for EBNA2 ((B, C) upper column, original magnification, 200x) or LMP1 ((B, C), lower column, original magnification, 200x) and **(D, E)** the quantification of EBNA2^+^ or LMP1^+^ cells/mm^2^ in splenic section of huNSG mice infected with either **(B, D, E)** 10^5^ RIU of wt, 3AKO or 3CKO 5 weeks p.i. (n = 17-20/group) indicated by black bars, **(D, E, G)** 10^5^ RIU of 3AKO or 3CKO 12 weeks p.i. (n = 3-5/group) indicated by red bars/square or **(C-E)** 10^6^ RIU of 3AKO or 3CKO 6 weeks p.i. (n = 11-13/group) or non-infected control (mock) huNSG mice. **(F, G)** Staining for EBNA1 (original magnification, 200x) in splenic sections of huNSG mice infected with 3AKO or 3CKO 12 weeks p.i. or non-infected control (mock) huNSG mice. **(A, D, E)** Pooled data from 4 and 2 experiments represented with the mean ± SEM, **P < 0.01, ***P < 0.001, Mann-Whitney U test.

Since lytic replication was not likely responsible for 3AKO and 3CKO persistence, we aimed to investigate the role of latent genes during infection with these viruses in vivo. Specifically, we explored three important proteins for B cell transformation upon EBV infection, the early transactivator EBNA2 and the later expressed LMP1 and LMP2A [10, 16]. In splenic B cells derived from huNSG mice infected with 3AKO or 3CKO, *EBNA2* mRNA expression was similar to wt LCLs cultured in vitro (Fig 5A). However, *LMP1* and *LMP2A* expression was significantly lower in both 3AKO and 3CKO infected animals compared to LCLs (Fig 5A). Furthermore, EBNA2^+^ cells were also detected by immunohistochemistry in splenic sections from 3AKO and 3CKO infected mice (Fig 5B and 5C), whereas LMP1 protein expression was virtually absent after inoculation with 10^5^ (Fig 5B) or 10^6^ RIU (Fig 5C). In line with the quantification of viral DNA in the spleen, there were significantly fewer EBNA2 and barely detectable LMP1 expressing cells in mice infected with 3AKO or 3CKO in comparison to mice infected with wt virus (Fig 5D and 5E). Interestingly, EBNA2 expression decreased with time from 5–6 weeks to 12 weeks of persistent infection with 3AKO or 3CKO (Fig 5D, red bars), while LMP1 expression remained low to almost non-detectable (Fig 5E, red bars). A single 3CKO infected mouse showed EBNA2 and LMP1 protein expression which was comparable to wt infection at 12 weeks after infection (Fig 5D and 5E, red square and Fig 5G). Therefore, in the majority of cases it seems that EBV lacking EBNA3A or EBNA3C persists with EBNA2 expression, but dramatically reduced or absent LMP1 and LMP2A expression, and seems to transit to latency 0/I after three months of EBV infection. To better characterize the latency stage at 12 weeks after infection, we determined EBNA1 protein expression by immunohistochemistry (Fig 5F and 5G). Whereas no EBNA1^+^ cells were detected in the majority of splenic sections (Fig 5F), the one 3CKO infected mouse showing EBNA2 and LMP1 expression also showed clear EBNA1 expression (Fig 5G). Therefore, 3AKO or 3CKO infection transits into latency 0 at 12 weeks after infection, but transition into a growth transforming latency program rarely occurs. Since only a subset of splenic B cells is infected with the recombinant 3AKO or 3CKO viruses, but the EBNA2 transcription levels are similar to homogenously EBV transformed LCLs, EBNA2 transcription seems to even be elevated, possibly as a compensatory mechanism for the diminished expression of LMP1 and 2. Nevertheless, this primarily EBNA2 driven proliferation allowed for the transition into mostly latency 0 after three months of infection.

### CD40 stimulation rescues EBNA3A and EBNA3C deficient EBV infected B cells in vitro

**Fig 6:**
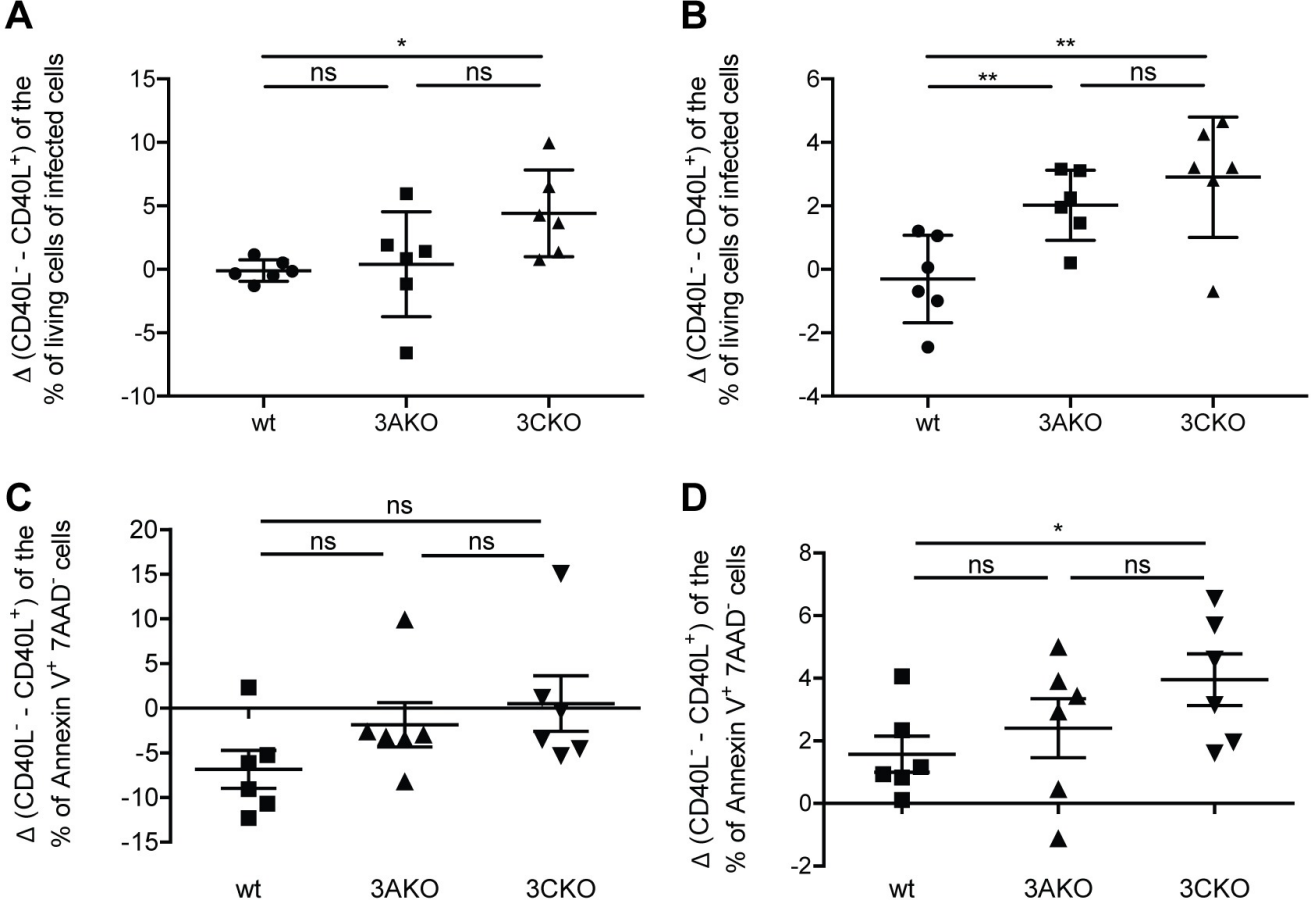
CD40 stimulation rescues EBNA3A and EBNA3C deficient EBV infected cells in vitro. Purified human B cells were infected with wt, 3AKO or 3CKO and cultured with irradiated fibroblasts either expressing CD40L or not (n = 6). **(A, B)** The change in the frequency of living infected cells (GFP^+^) cells between the culture condition with CD40L or without CD40L for wt, 3AKO or 3CKO infected B cells **(A)** 2 and **(B)** 3 weeks p.i., determined with 7AAD life-dead stain by flow cytometry. **(C, D)** The change in the frequency of early apoptotic Annexin V^+^ 7AAD^-^ cells between the culture condition with CD40L or without CD40L for wt, 3AKO or 3CKO infected B cells **(C)** 2 and **(D)** 3 weeks p.i., determined by flow cytometry. **(A-D)** Pooled data from 3 experiments represented with the mean ± SEM, *P < 0.05, **P < 0.01, ***P < 0.001, paired t-test.

Previous work has suggested that in the absence of LMP1 EBV can persist through CD40 stimulation provided from the microenvironment [28]. To address the role of CD40 stimulation in EBV lacking EBNA3A or EBNA3C, we infected CD19^+^ B cells, purified from human leucocyte concentrates with wt, 3AKO or 3CKO viruses in the presence of fibroblasts either expressing CD40L [29] or not. The frequency of living infected cells (EBV encoded GFP^+^) in the presence of CD40L (as inferred from the difference in culture conditions with or without CD40L) was significantly higher in 3CKO infected B cells 2 and 3 weeks p.i. as well as in 3AKO infected B cells 3 weeks p.i. compared to wt infected B cells (Fig 6A and 6B). Consistent with these findings, the frequency of Annexin V^+^ 7AAD^-^ early apoptotic cells in the absence of CD40L (as inferred from the difference in culture conditions with or without CD40L) were significantly increased after 3, but not 2 weeks in 3CKO infected cultures compared to wt infected cultures (Fig 6C and 6D). This data indicates that CD40 stimulation can markedly contribute to the persistence of 3AKO and 3CKO infected B cells and might be provided by the microenvironment for EBV persistence in the absence of EBNA3A and EBNA3C in vivo.

In order to assess the role of CD4^+^ T cells in providing a CD40 stimulating microenvironment as well as in exerting immune control in vivo, we depleted these cells with a CD4 specific antibody during 3AKO and 3CKO infection (S6 Fig). We observed elevated viral loads for 3AKO infection in spleen, lymph nodes and blood, while 3CKO viral loads were unchanged. This suggests that during EBV infection in the absence of EBNA3A, immune control by CD4^+^ T cells dominated over any supportive role of helper T cells, while these nurturing and immune control functions might have balanced each other during 3CKO infection and thereby viral loads were not altered upon CD4 depletion. Together, these data indicate that 3AKO and in particular 3CKO infection might benefit from CD40 stimulation for persistence.

### Elevated p16^INK4a^ expression in EBV infected cells without EBNA3A or 3C in vivo

**Fig 7:**
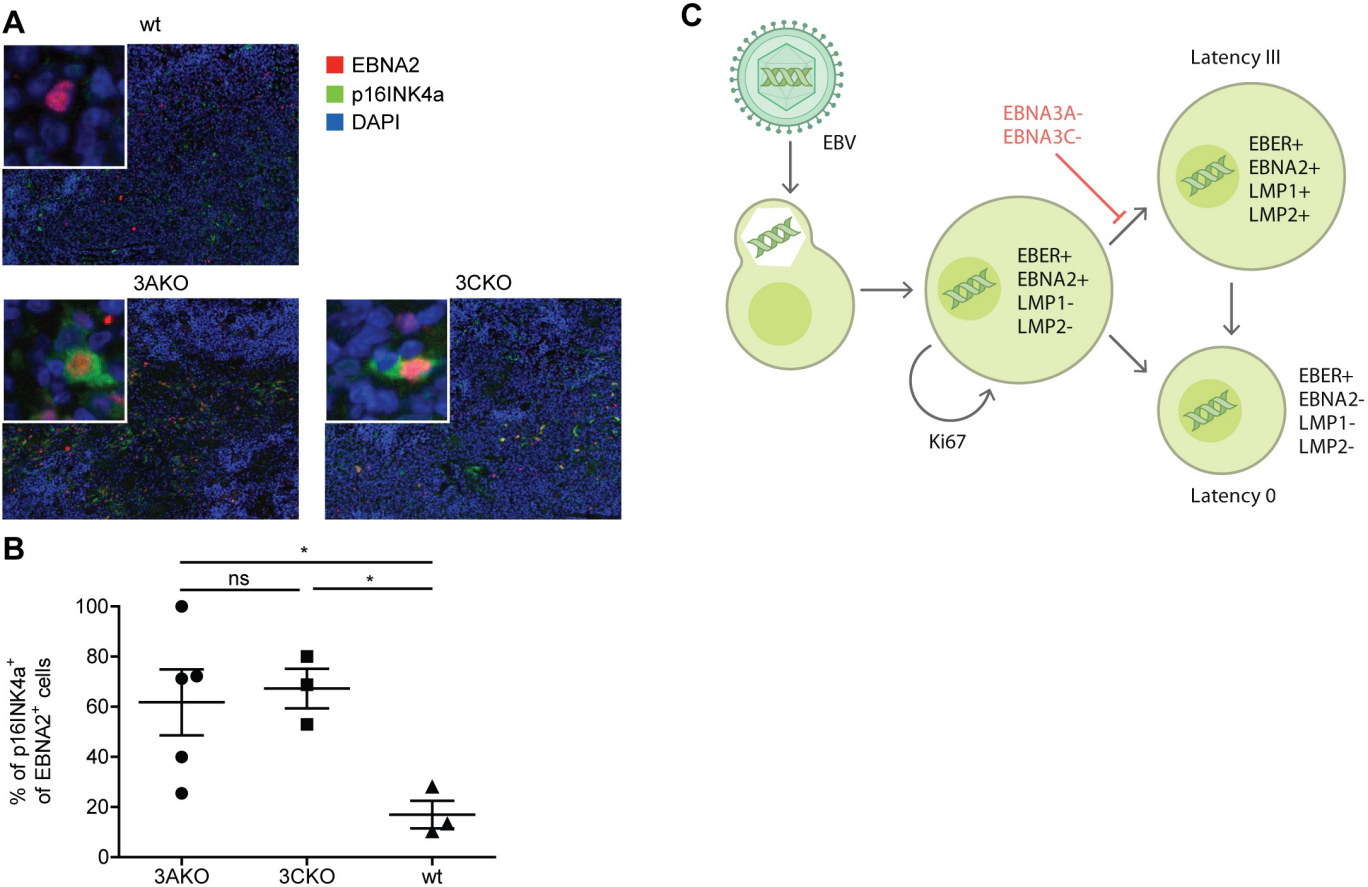
Elevated p16^INK4a^ expression in EBV infected cells without EBNA3A or 3C in vivo. **(A)** Representative immunofluorescence staining for EBNA2 (red), p16^INK4a^ (green) and DAPI (blue) (original magnification, 200x) in splenic sections of huNSG mice infected with wt, 3AKO or 3CKO 5 or 6 weeks p.i.. **(B)** Quantification of **A** with the frequency of p16^INK4a+^ EBNA2^+^ cells of all EBNA2^+^ cells (n = 3-5/group). Pooled data from 2 experiments with mean ± SEM, unpaired t-test with Welch's correction. **(C)** Schematic representation of B cell infection by EBV in humanized mice.

In order to determine if such a microenvironment support might be associated with cell cycle regulation in vivo, we monitored the p16^INK4a^ expression levels in EBNA2 positive cells in splenic sections of 3AKO or 3CKO infected mice 5 to 6 weeks p.i. by immunofluorescence (Fig 7). p16^INK4a^ and BIM expression levels had previously been reported to be suppressed by EBNA3A and 3C, thereby supporting proliferation and inhibiting cell death of EBV infected cells in vitro [10–13]. In contrast to wt infection most 3AKO and 3CKO infected cells expressed p16^INK4a^ (Fig 7B). Thus, absence of EBNA3A or 3C indeed elevates p16^INK4a^ levels in EBNA2 positive cells and presumably increases apoptosis risk of EBV infected B cells also in vivo. We propose that elevated p16^INK4a^ levels hinders efficient viral amplification and establishment of full latency III diverting infected cells into latency 0 for persistence. This further suggests that up-regulation of p16^INK4a^ in 3AKO and 3CKO infection might contribute to the lack of tumor formation that is observed in vivo.

## Discussion

Contrary to previous in vitro studies that require B cell transformation to maintain infected cells [14], we demonstrate that EBV infection can persist in the absence of EBNA3A or EBNA3C for at least 3 months in vivo. Persistence is mainly restricted to secondary lymphoid tissues and is characterized by initial EBNA2 expression with diminished LMP1 as well as LMP2A presence and lytic replication, while viral DNA in peripheral blood remains mostly undetectable. After 3 months solely EBER expression without expression of EBNA1, EBNA2 or LMP1 can be detected in EBV infected splenocytes. Since EBV infected B cells can be sustained in vitro in the absence of EBNA3A and EBNA3C by CD40L mediated CD40 engagement, we suggest that the microenvironment of secondary lymphoid tissues, possibly via CD40L on CD4^+^ T cells, allows for the persistence of EBNA3A and EBNA3C deficient EBV after infection. With this microenvironmental help and without the need to enter into complete latency III, EBV infected B cells seem to be able to access a viral gene expression program that is associated with long-term persistence in healthy EBV carriers and characterized by absence of viral protein expression and presence of non-translated viral RNAs like EBERs.

Indeed, absence of LMP1, which might directly result from loss of LMP1 promotor activation by EBNA3C [30–33] and constitutively engages the CD40 signaling machinery [34], has been previously reported to still allow EBV persistence in huNSG mice, even so no B cell transformation can be achieved with LMP1 deficient EBV in vitro [28]. The authors demonstrated that CD4^+^ T cells contributed via CD40L mediated CD40 stimulation to persistence and lymphomagenesis of LMP1 deficient EBV in vivo. Accordingly, T cells heavily infiltrated the respective EBNA2 positive tumors, which also expressed the EBNA2 driven c-myc oncogene. Therefore, the microenvironment in secondary lymphoid tissues can supplement signals that are essential for B cell transformation and persistence by EBV. We now demonstrate for an even earlier check-point in B cell transformation, EBNA3A or EBNA3C, which rescues B cells that are driven into proliferation by EBNA2 expression [10–12], that the microenvironment of secondary lymphoid tissues in huNSG mice allows EBV persistence in the absence of EBNA3A or EBNA3C expression. These findings argue that latency 0, which is considered to form the basis of EBV persistence in memory B cells of healthy virus carriers, can develop from an EBNA2 driven proliferation program without expression of EBNA3A, EBNA3C, LMP1 and LMP2A. Thus, latency III and 0 might represent two alternative pathways of infection originating from early latent EBV protein expression (Fig 7C).

Indeed, EBV persists in memory B cells at a frequency of 1 in 10^4^ to 10^5^ in the peripheral blood of healthy virus carriers without the expression of EBNA3A, EBNA3B, LMP1 and LMP2A [5, 7]. This cellular compartment of EBV infected memory B cells expands during symptomatic primary EBV infection, called infectious mononucleosis (IM), resulting in up to 50% being EBV positive [35]. Two pathways have been suggested to seed this peripheral memory B cell compartment with EBV infected cells. In tonsils of healthy virus carriers naïve B cell infection might lead to latency III and force the respective cells into the germinal center reaction [5]. Cytokines in the germinal center reaction, secreted by follicular helper T cells, might even be required to exit latency III and down-modulate EBNA3A and 3C expression [19]. Finally, late latent EBV gene expression like LMP1 and LMP2A might rescue EBV infected centroblasts and centrocytes into the memory B cell compartment by mimicking B cell receptor and T cell help signaling [5]. In contrast, germinal centers are largely dissolved in IM tonsils [36]. Under these circumstances independent of a germinal center reaction, direct infection of memory B cells might seed this persistent lymphocyte compartment with EBV without the need of LMP1 and LMP2A expression [36]. Similarly in our huNSG mice, germinal center development is rudimentary [37, 38], and EBNA3A or 3C deficient EBV in this study gained access to persistence in latency 0 without significant LMP1 and LMP2A expression. These considerations suggest that B cell transformation, which is only achieved on very rare occasions by EBNA3A and 3C deficient EBV viruses, is not necessary to develop the latency 0 program of long-term persistence in memory B cells and a direct differentiation from EBNA2 induced proliferation into persistence without viral protein expression can be achieved, bypassing the germinal center reaction.

## Materials and methods

### Recombinant EBV

Epstein-Barr-virus B95.8 (EBV) was produced from EBNA3A knock-out (3AKO), EBNA3C knock-out (3CKO) or wildtype (wt) BACs maintained in human embryonic kidney HEK293 cells (ATCC, Manassas, VA, USA) as described elsewhere [39]. The GFP expressing virus was titrated on Raji cells (ATCC, Manassas, VA, USA) in serial dilution and the GFP-expressing cells were analyzed by flow cytometry 2 days later to calculate Raji-infectious units (RIU).

### Mice with reconstituted human immune system components and infection with EBV

NOD-*scid* γ_c_^null^ (NSG) mice and HLA-A2 transgenic NSG were obtained from the Jackson Laboratories and maintained under specific pathogen-free conditions. Newborn mice (1–5 days old) were irradiated with 1 Gy using a Cs or x-ray source and after 5 to 7 hours reconstituted intrahepatically with 1–3 x 10^5^ CD34^+^ human hematopoietic progenitor cells (HPCs) purified from human fetal liver (HFL) tissue obtained from Advanced Bioscience Resources. Preparation of HFL tissue and isolation of human CD34^+^ cells were performed as described previously [18, 24]. Human immune component reconstitution levels were checked 10–12 weeks later and again just before the start of the experiments by flow cytometric immune phenotyping of PBMCs as previously described [40]. Mice were infected intraperitoneally with 1 × 10^5^ RIU of EBV wt, 3AKO or 3CKO or with 1 x 10^6^ RIU of EBV 3AKO or 3CKO and followed for 5 to 6 or 12 weeks respectively.

### CD4^+^ cell depletion

CD4^+^ cells were depleted with 100 μg of monoclonal antibody against human CD4 (clone OKT-4; Bio X Cell) via intraperitoneal inoculation for 3 consecutive days just prior to EBV infection. To deplete CD4^+^ cells for the duration of 5 weeks, the same injection procedure was repeated every 2 weeks.

### Quantification of viral load

Splenic tissue was processed using a QIAamp DNA Tissue Kit (QIAGEN) and total DNA from whole blood was extracted using the NucliSENS EasyMAG System (bioMérieux), both according to the manufacturer’s recommendations. TaqMan (Applied Biosystems) real-time PCR was used to quantify EBV DNA as previously described [41], with modified primers for the BamH1 W fragment (5′-CTTCTCAGTCCAGCGCGTTT-3′ and 5′-CAGTGGTCCCCCTCCCTAGA-3′) and a fluorogenic probe (5′-FAM CGTAAGCCAGACAGCAGCCAATTGTCAG-TAMRA-3′). All PCRs were run on an ABI Prism 7700 Sequence Detector (Applied Biosystems), and samples were analyzed in duplicates. Samples below the lower limit of quantification (LLOQ) of 67 EBV DNA copies were considered negative for EBV DNA. EBV wt infected mice without any sign of EBV infection were excluded from the analysis.

### Flow cytometry

All fluorescently labeled antibodies were purchased from BD Biosciences, BioLegend and Invitrogen (Thermo Fisher Scientific) (S1 Table). Lysis of erythrocytes in whole blood was done with NH_4_Cl. Spleens were mechanically disrupted and filtered through a 70 μm cell strainer before separation of mononuclear cells on Ficoll-Paque gradients. Cell suspensions were stained with antibodies for 30 min on ice, washed, and analyzed on FACSCanto or LSR Fortessa cytometers (BD Biosciences). Analysis of flow cytometric data was performed with FlowJo (Tree Star). The absolute numbers of leukocytes in each category were computed from the white blood cell count measured with a Beckman Coulter AcT diff Analyzer. Early apoptotic cells were assessed by incubating cells with AF647 annexin V (BioLegend) and 7AAD (BioLegend) diluted in annexin binding buffer for 15 min at room temperature in the dark, 400 μl binding buffer was added and the staining was directly analyzed on FACSCanto.

### Histology, immunohistochemistry and immunofluorescence

Tissue was fixed in 10% saline buffered formalin and paraffin embedded. For immunohistochemistry and immunofluorescence, 3 μm sections were processed on a Leica BOND-MAX or Bond-III automated immunohistochemistry system. Stainings were performed with monoclonal mouse anti-EBNA2 (clone PE2, Abcam), mouse anti-LMP1 (clone CS.1-4, Novocastra), mouse anti-BZLF1 (clone BZ1, Santa Cruz), mouse anti-CD138 (B-A38, Sigma-Aldrich), rat anti-EBNA1 (clone 1H4, kind gift from Dr. Kremmer, Munich, Germany), rabbit anti-Ki67 (clone SP6, Cell Marque), rabbit anti-p16^INK4a^ (clone EPR1473, Abcam), rabbit anti-CD3 (clone SP7, Diagnostic Biosystem), mouse anti-CD20 (clone L26, Dako) and rabbit anti-CD20 (Clone SP32, Cell Marque Lifescreen Ltd.). For immunofluorescence, the AF488 donkey anti-rabbit IgG (Jackson ImmunoResearch), Cy3 donkey anti-mouse IgG (Jackson ImmunoResearch), DyLight549 horse anti-mouse IgG (Vectrolabs) and DAPI (Sigma) were used. In situ hybridization (ISH) for EBV-encoded RNAs (EBER-ISH) was done as described previously, employing diaminobenzidine (DAB) chromogen (Zytomed Systems, Berlin, Germany) as substrate [42]. Quantification of BZLF1, EBER and EBER plus CD20 was performed as described previously for quantitative evaluation of labeled cells [43]. The number of labeled cells was determined per 1mm^2^ using the image analysis software HISTO (Biomas). EBNA2, LMP1 and CD138 stainings, EBER ISH as well as EBNA2 plus CD3, EBNA2 plus CD20, EBNA2 plus Ki67 and EBNA2 plus p16^INK4a^ co-stainings were analyzed with a Vectra3 automated quantitative pathology imaging system (PerkinElmer) using Vectra and InForm software analysis (PerkinElmer). Snapshots were taken using a scanning protocol created with InForm tissue segmentation to recognize the tissue. Briefly, the stained slides were loaded onto the Vectra slide scanner, 1 to 10 images with 20 × objective lens (0.5 micron/pixel) were taken with a CCD using the scanning protocol. Images taken were used to set up an algorithm in InForm to recognize and count EBNA2, LMP1 or CD138 positive cells, respectively. Signal cross-talk was eliminated using one chromogen only (DAB, hematoxylin or fast red) control stains. If the algorithm was >90% confident that the signal was positive, the cells were counted and the number of positive cells per 1mm^2^ was determined. InForm was also used to set up an algorithm to calculate the percentage of EBNA2 plus Ki67, EBNA2 plus CD20 and EBNA2 plus CD3 double positive cells of all EBNA2 positive cells. The percentage of EBNA2 and p16^INK4a^ double positive cells of all EBNA2 positive cells was quantified by an independent observer.

### B cell isolation from infected mice or human leucocyte concentrates

Human B cells were isolated from spleen suspensions of infected mice or from human leucocyte concentrates (blood bank Zurich) using CD19 microbeads (Miltenyi Biotec) according to the manufacturer’s recommendations.

### In vitro infection of human B cells with wt, 3AKO or 3CKO EBV

10^6^ magnetically sorted human B cells from human leukocyte concentrates were infected with wt, 3AKO or 3CKO EBV with the addition of irradiated (140Gy) 3T3 fibroblast expressing CD40L or not [29]. Irradiated fibroblasts were replaced weekly. The cells were maintained in RPMI 1640 medium (Gibco, Thermo Fisher Scientific) with 10% FCS, streptomycin and penicillin. Analysis of living cells was done using flow cytometry 14 and 21 days p.i..

### qPCR for viral gene expression

RNA was isolated using the RNeasy Mini Kit (QIAGEN) according to the manufacturer’s recommendations either of the magnetically enriched CD19^+^ fraction of infected mice or infected buffy coats. To remove contaminating genomic DNA, a 15min on-column DNAse treatment was included during the RNA isolation (RNAse-Free DNAse Set, Qiagen). Purified RNA was reverse transcribed using GoScript Reverse Transcriptase (Promega) for 1h at 42°C according to the recommendations of the manufacturer using a primer mix combining previously described gene-specific RT primers [44] at concentrations of 10μM each. cDNA was used to amplify EBNA2, LMP1, LMP2A, BZLF1 and GAPDH using previously published primers with 5’FAM/3’TAMRA [44], equal RNA input and Taqman universal PCR reagents (Applied Biosystems). qPCR was performed in triplicates on a C1000 Touch CFX384 Real-Time platform (Bio-Rad, Hercules, CA, USA) as described elsewhere [45]. Cq values were determined with the CFX-manager software (Biorad) and relative mRNA levels were normalized to the reference gene GAPDH. The detection limits of the RT-qPCR assays was calculated with the background Cq value and the highest *GAPDH* expression found in mouse samples of the used experiments.

### Statistical analysis

Statistical analysis and graph preparation was performed with Prism software (GraphPad). Two-tailed Mann-Whitney U, unpaired two-tailed t-test with Welch's correction, two-way ANOVA tests with Bonferroni correction, paired t-test or Fisher’s exact test for actual numbers were used. A p value of < 0.05 was considered statistically significant.

### Ethics statement

All animal protocols were conducted according to the Swiss Animal Welfare Act, Tierschutzgesetz (TSchG) and approved by the veterinary office of the canton of Zurich, Switzerland (protocols 148/2011, 209/2014 and 159/17). All studies involving human samples were reviewed and approved by the cantonal ethics committee of Zurich, Switzerland (protocol KEK-StV-Nr.19/08). The used human samples were anonymized.

## Supporting information

S1 Fig EBV is mainly detected in B cells upon infection with 3AKO, 3CKO or wt viruses. Staining for **(A, B)** EBNA2 and CD20 or **(A, B)** EBNA2 and CD3 of splenic sections of huNSG mice infected with either **(A)** 10^5^ RIU of wt, 3AKO or 3CKO 5 weeks p.i. or **(B)** 10^6^ RIU of 3AKO or 3CKO 6 weeks p.i.. **(C)** Frequency of EBNA2^+^ cells with or without CD20 or CD3 expression in mice infected with either 10^5^ RIU of wt, 3AKO or 3CKO 5 weeks p.i. or 10^6^ RIU of 3AKO or 3CKO. **(D)** EBER ISH and CD20 staining of splenic section of huNSG mice infected with either 10^5^ RIU of 3AKO or 3CKO 12 weeks p.i..
(TIF)

S2 Fig EBNA3A or EBNA3C deficient EBV infections show a trend toward CD8^+^ T cell expansion in peripheral blood. The change between the beginning and the end of the experiment (Δend-start) in the number of **(A)** blood CD8^+^ T cells / ml and **(B)** blood CD4^+^ T cells / ml of huNSG mice infected with either 10^5^ RIU of wt, 3AKO or 3CKO 5 weeks p.i. (n = 14-17/group) or 10^6^ RIU of 3AKO or 3CKO 6 weeks p.i. (n = 7-13/group) or non-infected control (mock) huNSG mice as determined by flow cytometry and white blood cell counting with a hemocytometer **(A, B)** Pooled data from 4 low and 2 high infectious dose experiments with the mean ± SEM. *P < 0.05, **P < 0.01, ***P < 0.001, by Mann-Whitney U test.
(TIF)

S3 Fig EBNA3A or EBNA3C deficient EBV infection causes T cell activation. The frequency of HLA-DR^+^ CD8^+^ **(A, C)** blood and splenic **(B, D)** T cells or HLA-DR^+^ CD4^+^ **(E, G)** blood and splenic **(F, H)** T cells of huNSG mice infected with either 10^5^ RIU of wt, 3AKO or 3CKO 5 weeks p.i. (n = 14-21/group) or 10^6^ RIU of 3AKO or 3CKO 6 weeks p.i. (n = 7-13/group) or non-infected control (mock) huNSG mice was determined by flow cytometry. **(A-H)** Pooled data from 4 low and 2 high infectious dose experiments with mean ± SEM. *P < 0.05, **P < 0.01, ***P < 0.001, Mann-Whitney U test.
(TIF)

S4 Fig EBNA3A and EBNA3C deficient EBV infected mice show reduced lytic replication. **(A)** Representative immunohistochemistry staining for CD138 (original magnification, 200x) and the **(B)** quantification of CD138^+^ cells/mm^2^ in splenic sections from huNSG mice infected with 10^6^ RIU of 3AKO or 3CKO at 6 weeks p.i. and from non-infected control (mock) huNSG mice. Pooled data from 2 experiments represented with the mean ± SEM, Mann-Whitney U test. **(C)** Representative immunohistochemistry staining for BZLF1 (original magnification, 400x) in splenic sections of huNSG mice infected with 10^6^ RIU of 3AKO or 3CKO 6 weeks p.i. and non-infected control (mock) huNSG mice.
(TIF)

S5 Fig EBNA3C deficient EBV shows reduced lytic replication in vitro. Relative mRNA expression of *BZLF1* normalized to *GAPDH* as determined by RT-qPCR in purified human CD19^+^ B cells infected with wt, 3AKO or 3CKO with or without irradiated fibroblasts (FB) either expressing CD40L or not 3 weeks p.i. (n = 6–13). Pooled data from 3 experiments represented with the mean ± SEM, *P < 0.05, **P < 0.01, ***P < 0.001, Mann-Whitney U test.
(TIF)

S6 Fig Elevated EBV loads in 3AKO infected mice upon CD4^+^ T cell depletion. **(A)** Splenic endpoint viral DNA load and **(B)** viral DNA load per gram lymph node tissue determined by qPCR of huNSG mice inoculated with a CD4 specific antibody and infected with 10^5^ RIU of 3AKO or 3CKO for 5 weeks (n = 4-6/group). Values for mice in which no viral DNA was detected are plotted on the X-axis. **(C)** Blood DNA viral load over time determined by qPCR of huNSG mice infected with 10^5^ RIU of 3AKO or 3CKO 5 weeks p.i. (n = 4-6/group). Horizontal dashed line indicates the viral load of 3 times the lower limit of quantification (LLOQ). Horizontal dotted line indicates the LLOQ. **(A-B)** Data from 1 experiment is displayed with geometric mean for splenic and lymph node viral load and SEM for blood viral load, *P < 0.05, **P < 0.01, ***P < 0.001, two-way ANOVA with Bonferroni correction for blood viral load and Mann-Whitney U test for splenic and lymph node viral load.
(TIF)

S1 Table Overview of fluorescently labeled antibodies used in this study for flow cytometry. (DOCX)
