# Supplementary figures and images for: EBV persistence without its EBNA3A and 3C oncogenes in vivo
Source: PLoS Pathog. 2018 Apr 30;14(4):e1007039. doi: 10.1371/journal.ppat.1007039 (PMC5945050; doi:10.1371/journal.ppat.1007039)

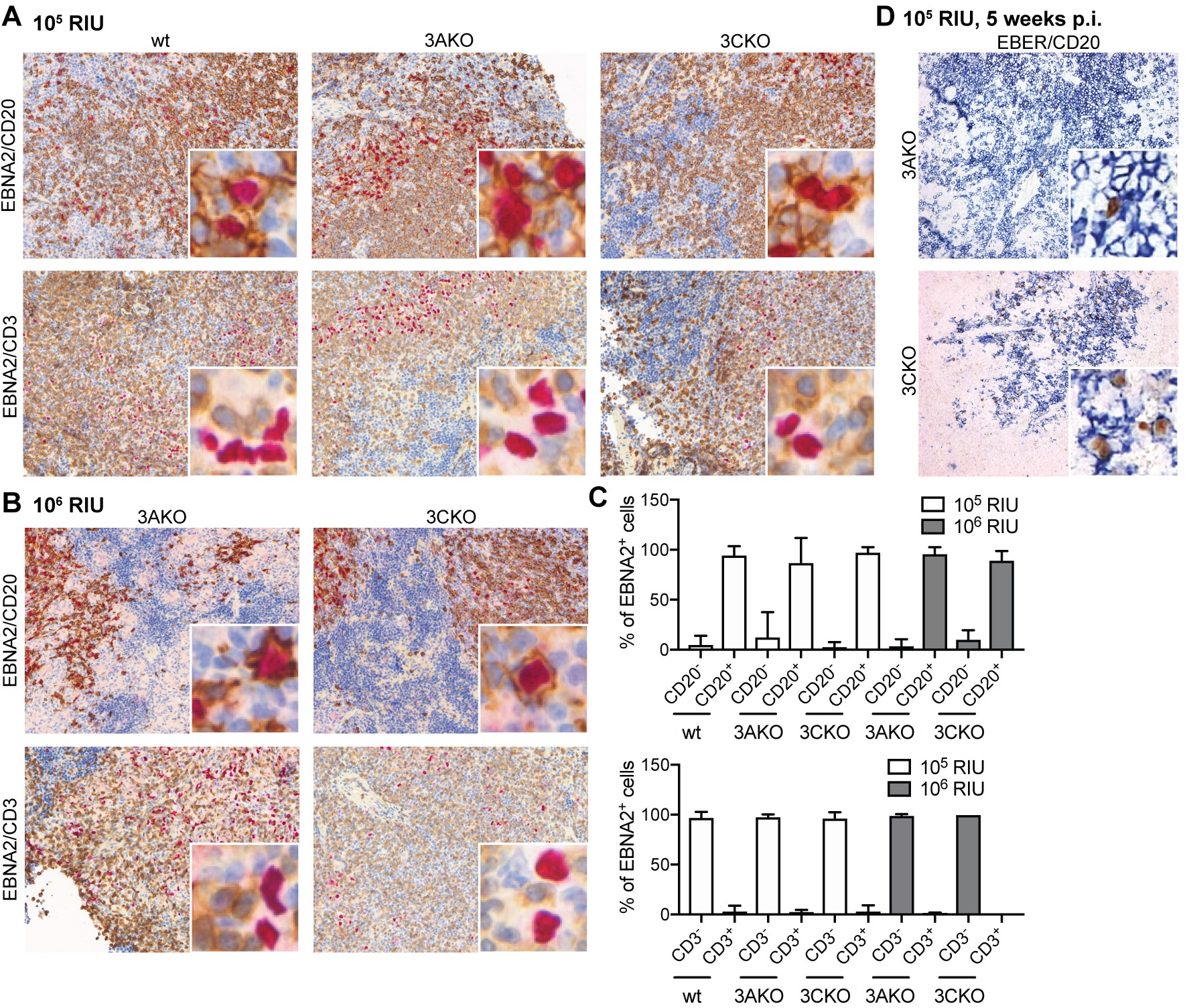

Supplement: S1 Fig — Staining for (A, B) EBNA2 and CD20 or (A, B) EBNA2 and CD3 of splenic sections of huNSG mice infected with either (A) 105 RIU of wt, 3AKO or 3CKO 5 weeks p.i. or (B) 106 RIU of 3AKO or 3CKO 6 weeks p.i.. (C) Frequency of EBNA2+ cells with or without CD20 or CD3 expression in mice infected with either 105 RIU of wt, 3AKO or 3CKO 5 weeks p.i. or 106 RIU of 3AKO or 3CKO. (D) EBER ISH and CD20 staining of splenic section of huNSG mice infected with either 105 RIU of 3AKO or 3CKO 12 weeks p.i.. (TIF) [file ppat.1007039.s001.tif]

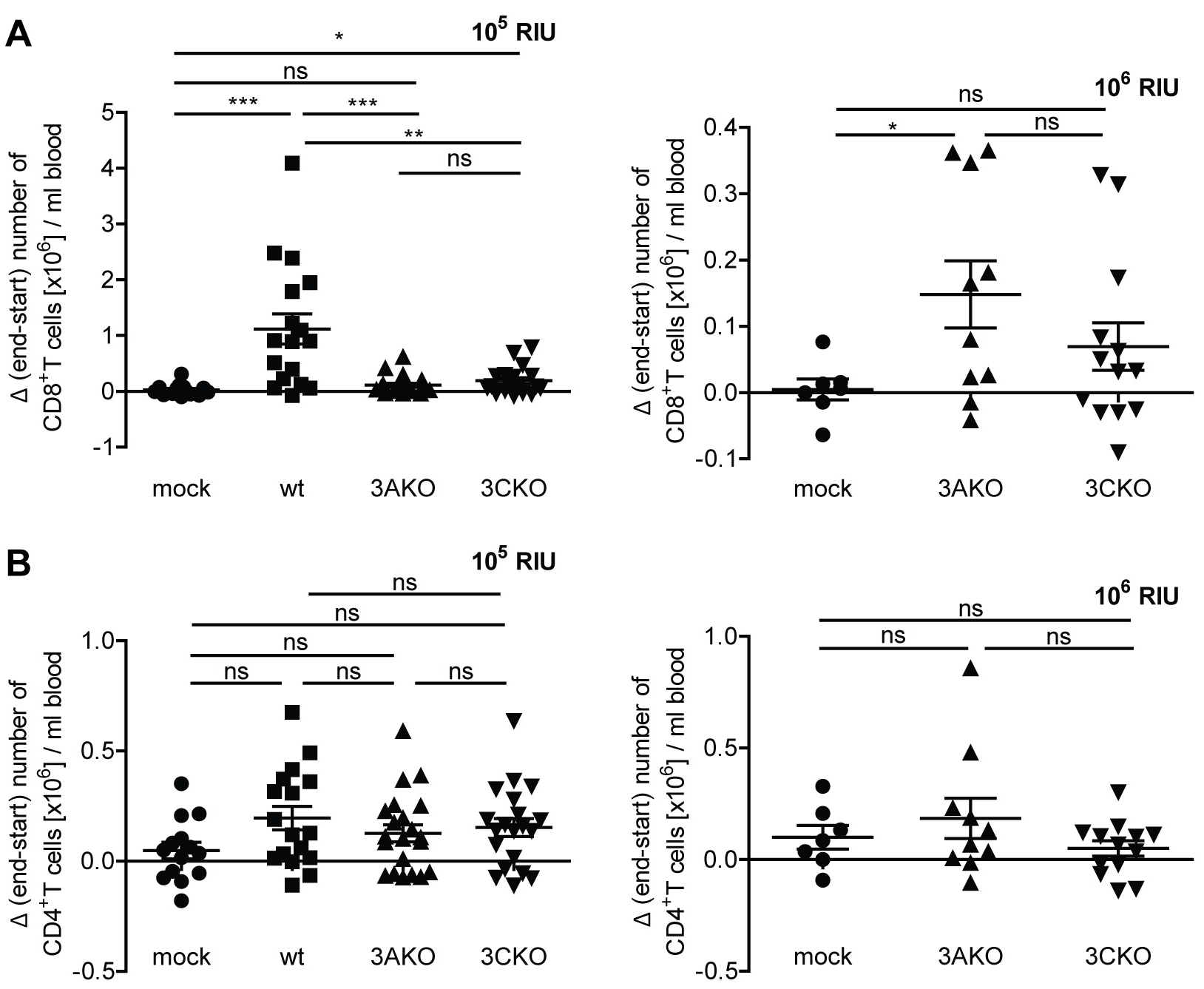

Supplement: S2 Fig — The change between the beginning and the end of the experiment (Δend-start) in the number of (A) blood CD8+ T cells / ml and (B) blood CD4+ T cells / ml of huNSG mice infected with either 105 RIU of wt, 3AKO or 3CKO 5 weeks p.i. (n = 14-17/group) or 106 RIU of 3AKO or 3CKO 6 weeks p.i. (n = 7-13/group) or non-infected control (mock) huNSG mice as determined by flow cytometry and white blood cell counting with a hemocytometer (A, B) Pooled data from 4 low and 2 high infectious dose experiments with the mean ± SEM. *P < 0.05, **P < 0.01, ***P < 0.001, by Mann-Whitney U test. (TIF) [file ppat.1007039.s002.tif]

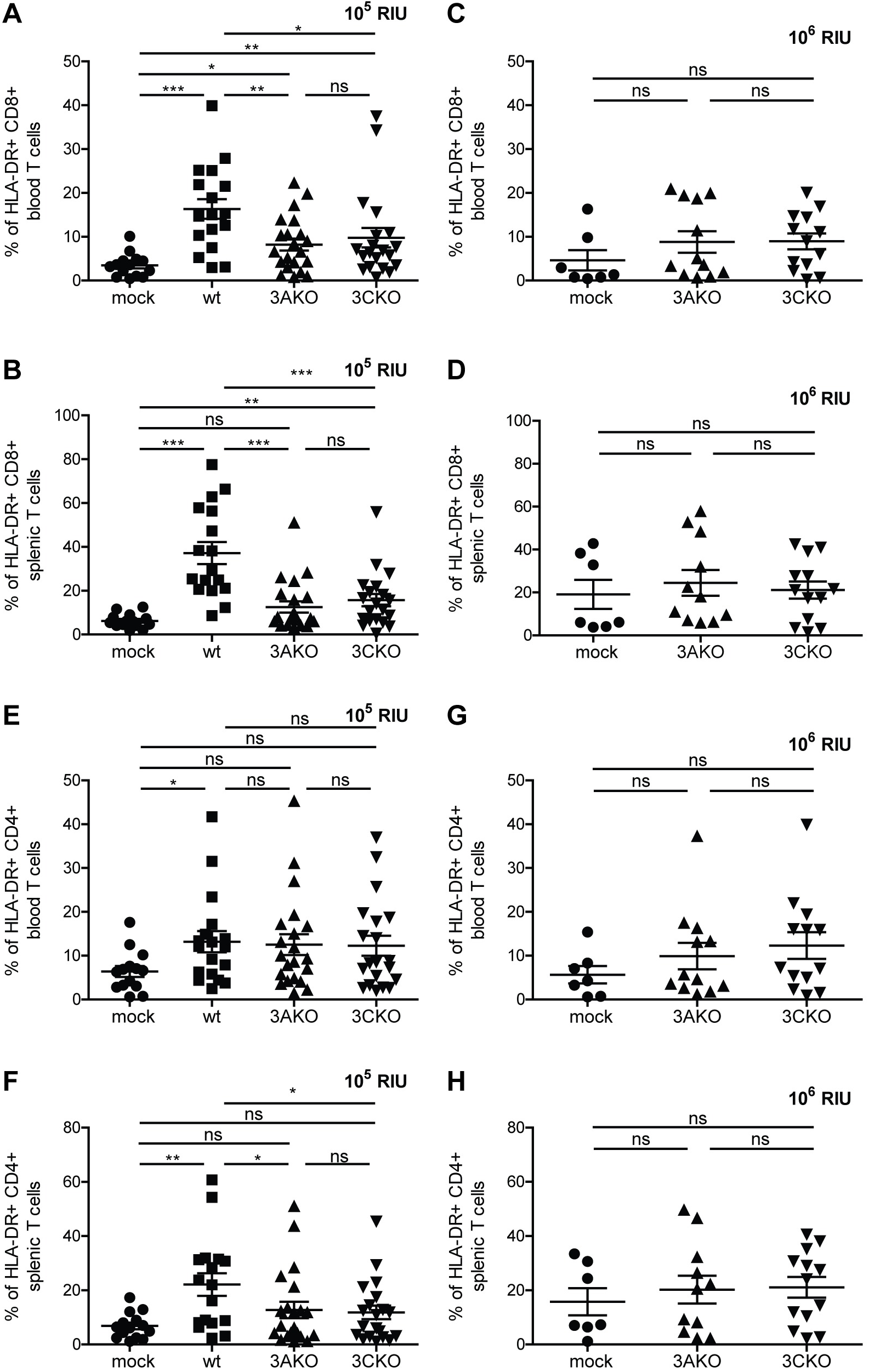

Supplement: S3 Fig — The frequency of HLA-DR+ CD8+ (A, C) blood and splenic (B, D) T cells or HLA-DR+ CD4+ (E, G) blood and splenic (F, H) T cells of huNSG mice infected with either 105 RIU of wt, 3AKO or 3CKO 5 weeks p.i. (n = 14-21/group) or 106 RIU of 3AKO or 3CKO 6 weeks p.i. (n = 7-13/group) or non-infected control (mock) huNSG mice was determined by flow cytometry. (A-H) Pooled data from 4 low and 2 high infectious dose experiments with mean ± SEM. *P < 0.05, **P < 0.01, ***P < 0.001, Mann-Whitney U test. (TIF) [file ppat.1007039.s003.tif]

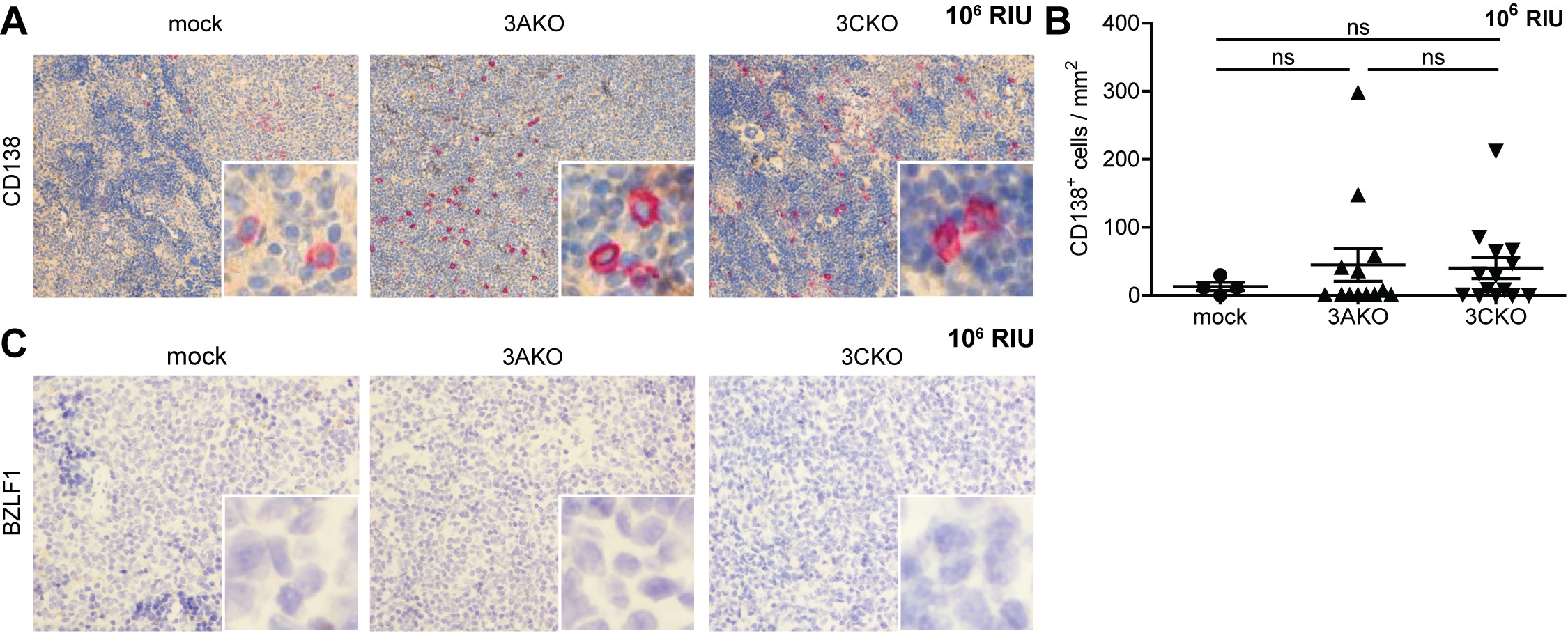

Supplement: S4 Fig — (A) Representative immunohistochemistry staining for CD138 (original magnification, 200x) and the (B) quantification of CD138+ cells/mm2 in splenic sections from huNSG mice infected with 106 RIU of 3AKO or 3CKO at 6 weeks p.i. and from non-infected control (mock) huNSG mice. Pooled data from 2 experiments represented with the mean ± SEM, Mann-Whitney U test. (C) Representative immunohistochemistry staining for BZLF1 (original magnification, 400x) in splenic sections of huNSG mice infected with 106 RIU of 3AKO or 3CKO 6 weeks p.i. and non-infected control (mock) huNSG mice. (TIF) [file ppat.1007039.s004.tif]

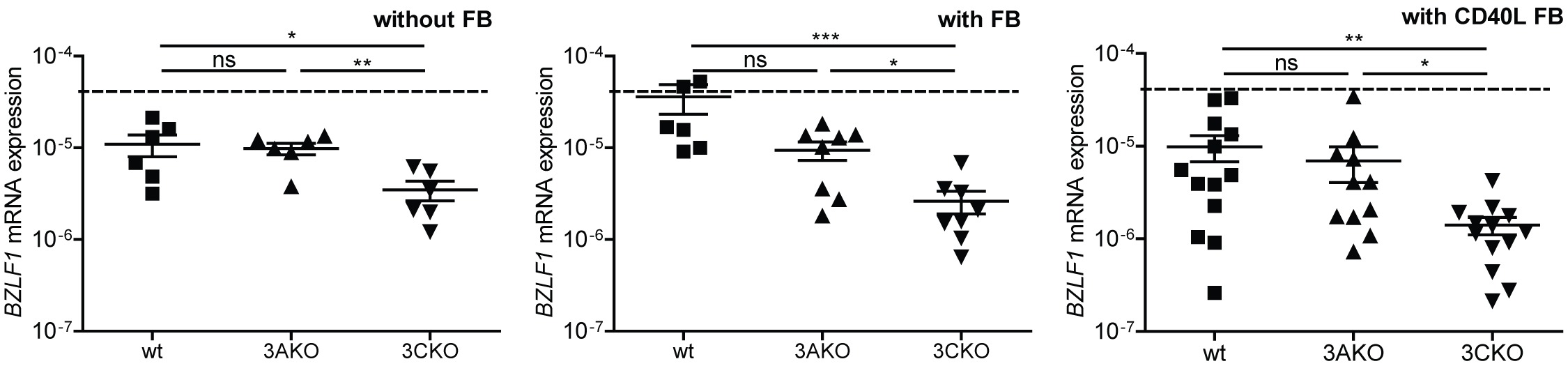

Supplement: S5 Fig — Relative mRNA expression of BZLF1 normalized to GAPDH as determined by RT-qPCR in purified human CD19+ B cells infected with wt, 3AKO or 3CKO with or without irradiated fibroblasts (FB) either expressing CD40L or not 3 weeks p.i. (n = 6–13). Pooled data from 3 experiments represented with the mean ± SEM, *P < 0.05, **P < 0.01, ***P < 0.001, Mann-Whitney U test. (TIF) [file ppat.1007039.s005.tif]

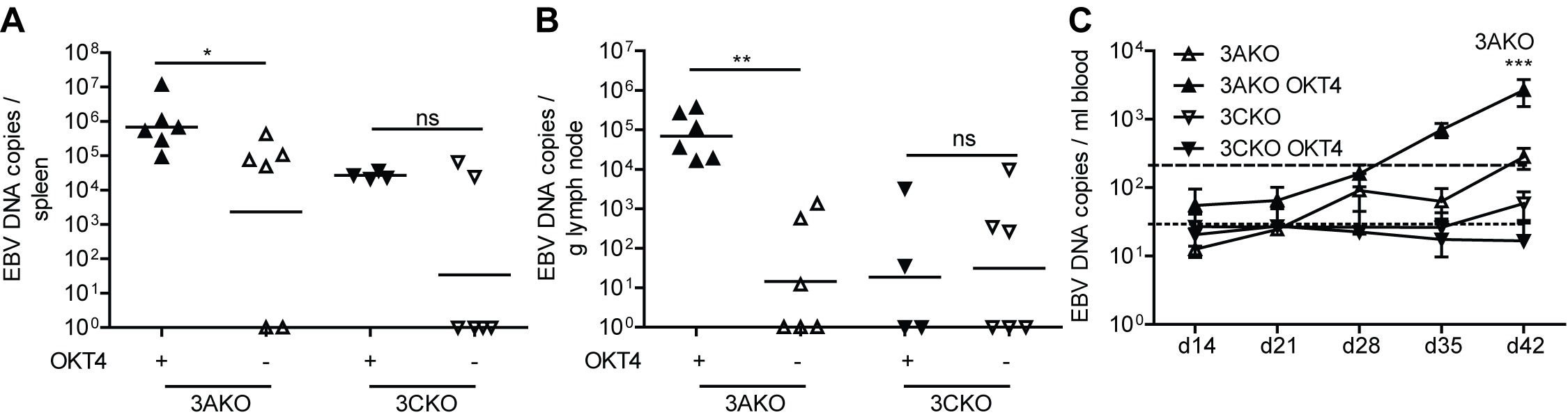

Supplement: S6 Fig — (A) Splenic endpoint viral DNA load and (B) viral DNA load per gram lymph node tissue determined by qPCR of huNSG mice inoculated with a CD4 specific antibody and infected with 105 RIU of 3AKO or 3CKO for 5 weeks (n = 4-6/group). Values for mice in which no viral DNA was detected are plotted on the X-axis. (C) Blood DNA viral load over time determined by qPCR of huNSG mice infected with 105 RIU of 3AKO or 3CKO 5 weeks p.i. (n = 4-6/group). Horizontal dashed line indicates the viral load of 3 times the lower limit of quantification (LLOQ). Horizontal dotted line indicates the LLOQ. (A-B) Data from 1 experiment is displayed with geometric mean for splenic and lymph node viral load and SEM for blood viral load, *P < 0.05, **P < 0.01, ***P < 0.001, two-way ANOVA with Bonferroni correction for blood viral load and Mann-Whitney U test for splenic and lymph node viral load. (TIF) [file ppat.1007039.s006.tif]
