# Supplementary material for: EBV persistence without its EBNA3A and 3C oncogenes in vivo
Source: PLoS Pathog. 2018 Apr 30;14(4):e1007039. doi: 10.1371/journal.ppat.1007039 (PMC5945050; doi:10.1371/journal.ppat.1007039)
Supplement: S1 Table — (DOCX) [file ppat.1007039.s007.docx]

**Supplemental Experimental Procedures**

**Table S1. Overview of fluorescently labeled antibodies for flow cytometry**

| Molecule | Clone | Fluorophore | Company |
| --- | --- | --- | --- |
| NKp46 | 9E2 | APC | BD Bioscience |
| CD19 | HIB19 | PE-Cy7 | BioLegend |
| CD3 | VCHT1 | PE | BioLegend |
| CD3 | VCHT1 | BV785 | BioLegend |
| CD4 | RPA-T4 | APC-Cy7 | BioLegend |
| CD4 | RPA-T4 | BV510 | BioLegend |
| CD45 | HI30 | Pacific Blue | BioLegend |
| CD45 | HI30 | BV605 | BioLegend |
| CD8 | SK1 | PerCP | BioLegend |
| HLA-DR | L243 | FITC | BioLegend |
| HLA-DR | L243 | Pe-Cy7 | BioLegend |
| CD19 | SJ25-C1 | PE Texas Red | Invitrogen |
